# From Search to Experience: Dynamic Reweighting of Evaluative Criteria in Experience-Based Decisions

**DOI:** 10.3390/bs16030340

**Published:** 2026-02-28

**Authors:** Zhen-Bang Zhong, Hong-Youl Ha

**Affiliations:** Department of International Trade, Dongguk University, Seoul 04620, Republic of Korea; zzb971024@gmail.com

**Keywords:** experience-based decision-making, evaluative judgment stabilization, selective reinforcement, satisfaction as cognitive signal, platform-mediated choice

## Abstract

Researchers typically treat platform loyalty in online travel agency (OTA) settings as a static outcome of satisfaction, even though repeated platform use unfolds over time. However, consumers update evaluative judgments through learning and memory as they move from pre-consumption expectations to post-consumption experiences, gradually stabilizing evaluations rather than continuously revising them. To address this gap, we use a two-wave time-lagged survey capturing pre- and post-consumption evaluations to examine when and how satisfaction-based platform loyalty strengthens in OTA-mediated hotel choice. The results show that the relationship between satisfaction and platform loyalty intentions intensifies after consumption. Satisfaction increasingly functions as a decision-guiding cognitive signal. This strengthening reflects experience-driven reweighting of hotel choice attributes. Consumers reweight existing criteria through experience rather than introducing new ones. Notably, the importance of core attributes, especially room quality and online reviews, increases as experience accumulates. Satisfaction and platform loyalty intentions also display significant carryover effects, indicating that prior evaluations shape subsequent judgments through memory-based continuity. By showing that evaluative judgments stabilize through selective reinforcement of existing criteria, this study explains how satisfaction transforms from an outcome judgment to a cognitive anchor for future decisions and underscores the value of longitudinal approaches for understanding early-stage experience-based decision dynamics.

## 1. Introduction

Recent advances in digital platforms raise fundamental questions about how individuals form, revise, and stabilize evaluative judgments in experience-based decision environments. Although research on online travel agencies (OTAs) has traditionally focused on market outcomes such as satisfaction and loyalty, a behavioral science perspective shifts attention to the cognitive processes through which evaluations evolve across sequential decisions. This perspective aligns closely with the decisions-from-experience literature, which shows that experiential learning, memory retrieval, and feedback from prior outcomes, rather than static preferences, shape repeated choices (Erev & Haruvy, 2013; Hertwig & Erev, 2009). Scholars increasingly reconsider what they once treated as a relatively stable relationship between service attributes, satisfaction, and loyalty in platform-mediated, information-rich, and experience-driven decision contexts. In this study, we examine the transition from pre-consumption expectations to post-consumption experience within a focal OTA-mediated decision episode.

In OTA settings, consumers do not make hotel choices as isolated events; they search, compare, book, experience, and re-evaluate options repeatedly over time. Therefore, repeated platform use provides a natural setting for examining how evaluative cognition unfolds across sequential choices. From a decisions-from-experience perspective, ongoing interactions allow individuals to learn from outcomes, selectively attend to previously successful cues, and rely on memory-based sampling when forming subsequent evaluations (Erev & Ert, 2023; Pessach et al., 2025). Thus, loyalty moves beyond behavioral outcomes; it becomes a stabilized evaluative judgment shaped by learning and memory across repeated decision episodes.

Building on this premise, this study conceptualizes OTA-mediated hotel choice as a dynamic consumption process in which individuals continually perceive, evaluate, and reprioritize hotel choice attributes as experience accumulates. OTAs facilitate extensive pre-consumption search and provide post-consumption feedback, such as ratings and reviews. Through repeated interaction with these features, consumers recalibrate expectations and reorganize the relative importance of evaluative cues. Consistent with experience-based learning theories, individuals reinforce attributes that demonstrated predictive reliability in prior decisions rather than continually expanding their criterion set (Roth et al., 2016). Instead of adding new criteria, consumers build decision confidence by adjusting the weight of existing criteria over time.

Although service research has long treated customer satisfaction as a cornerstone, most studies model it as a static outcome driven by isolated attributes measured at a single point in time (Albayrak & Caber, 2015; Qu et al., 2000; Srivastava & Kumar, 2021). Even research described as longitudinal often applies fixed-perspective models that fail to capture the cumulative and adaptive nature of platform-mediated decision experiences (Ha, 2012; Wu et al., 2012). As a result, scholars rarely theorize satisfaction as a dynamic cognitive judgment constructed, reinforced, and transmitted during the shift from pre-consumption expectations to post-consumption experience through learning and memory. This limitation matters particularly from a behavioral decision-making perspective because experience-based research suggests that individuals disproportionately rely on prior outcomes when forming subsequent judgments, which creates systematic carryover and reinforcement effects (Erev & Haruvy, 2013; Hertwig & Erev, 2009).

Despite the inherently iterative nature of OTA-mediated hotel choice, the literature says little about how evaluative criteria evolve. Prior studies primarily identify which attributes matter—price, location, ratings, or reviews—while assuming that their relative importance remains stable across repeated choices (Zhi & Ha, 2024). Researchers also model the satisfaction–loyalty relationship as time-invariant, overlooking the possibility that its role changes as experience accumulates. From a behavioral science standpoint, satisfaction may increasingly operate as a judgment-guiding signal as evaluative criteria narrow and stabilize. Decisions-from-experience research suggests that repeated exposure to outcomes leads individuals to rely more heavily on learned evaluative signals, which then serve as cognitive shortcuts in future decisions (Erev & Ert, 2023; Pessach et al., 2025).

This omission has important theoretical implications. Treating OTA-mediated hotel choice as independent decisions obscures its nature as a learning-based consumption system characterized by expectation adjustment and cumulative evaluation. Static models risk misrepresenting evaluative cognition in repeated-choice environments, where earlier judgments systematically shape later ones through memory continuity. This limitation restricts researchers from explaining when and why certain cues become dominant or peripheral over time. Experience-based decision theory suggests that individuals reinforce previously diagnostic attributes and attenuate less informative ones as experience grows.

In platform-mediated environments such as OTAs, researchers have underexplored how consumers attribute service outcomes to the platform. Although hotels deliver service attributes, consumers also evaluate whether the platform’s information, such as reviews, ratings, and recommendations, accurately predicted the experienced quality. When the platform facilitates successful choices and reduces search uncertainty, consumers may attribute positive service outcomes to the platform, thereby strengthening platform loyalty.

Addressing this gap becomes especially important in repeated, platform-mediated decision environments such as OTAs, where researchers can observe pre-consumption expectations and post-consumption evaluations within the same choice system. Repeated bookings generate observable shifts in attribute salience, satisfaction, and loyalty that cross-sectional designs cannot capture (Su & Ha, 2025). By adopting a longitudinal perspective, researchers can move beyond identifying which attributes matter and instead explain when and how their influence changes. From this perspective, time reorganizes and stabilizes evaluative structures; it is not merely a before-and-after comparison. This temporal dimension is central to understanding experience-based decision-making, where individuals learn through sequential feedback and evaluative updating.

To address these issues, the present study adopts a longitudinal design to examine how evaluative criteria evolve in OTA-mediated hotel booking experiences and how this evolution reshapes the satisfaction–loyalty relationship over time. We focus on the transition from pre-consumption expectations to post-consumption experience within a focal OTA-mediated decision episode. Drawing on the consumption system framework (Mittal et al., 1999), Oliver’s satisfaction-based loyalty theory (Oliver, 2010), and insights from the decisions-from-experience literature, we conceptualize hotel choice attributes as time-varying predictors whose influence on satisfaction and loyalty shifts through experiential learning. Specifically, we investigate (1) how the relative importance of evaluative criteria shifts over time and (2) how such shifts transform satisfaction from an outcome judgment into a decision-guiding cognitive signal during the expectation–experience transition.

This study makes two key contributions to behavioral decision research. First, it shows that evaluative judgments begin to stabilize through experience-based updating rather than growing increasingly complex. By linking attribute reweighting to experiential learning, the study extends decisions-from-experience research into platform-mediated consumption contexts and demonstrates how experiential feedback reorganizes evaluative criteria. By documenting attribute reweighting rather than attribute expansion, the study clarifies how evaluative cognition simplifies and consolidates through experience. Second, the study identifies carryover effects in satisfaction and platform loyalty intentions, highlighting the role of memory-based continuity in repeated decisions. Prior judgments persist and shape future evaluations, providing empirical support for the central proposition of decisions-from-experience research—that individuals rely on past outcomes to guide future decisions—within a real-world platform setting.

Together, these findings underscore the importance of longitudinal approaches for explaining how evaluative cognition stabilizes and guides behavior in experience-based decision contexts, including online travel platforms. By integrating research on the dynamic nature of satisfaction and loyalty, social-psychological theories of attitude stability and memory-based evaluation, and platform-mediated decision-making, this study offers a more comprehensive account of how evaluative processes evolve in digital service environments.

## 2. Literature Review and Theoretical Framework

This study grounds its arguments in established theoretical frameworks that collectively justify examining dynamic consumer evaluations over time. From a behavioral science perspective, these frameworks converge on the idea that repeated decisions involve adaptive cognitive processes rather than static preference structures. Drawing on general living systems theory (Reidenbach & Oliva, 1981), we conceptualize OTA-mediated hotel choice as an adaptive, platform-based consumption system organized around an ongoing cycle of information search, platform-facilitated evaluation, consumption experience, and behavioral feedback that evolves through repeated interactions. Within this system, consumers update their evaluations based on accumulated OTA-mediated experiences, which generate dynamic shifts in satisfaction and platform loyalty intentions over time. This systemic lens frames consumer behavior as a learning-driven process in which individuals revise evaluative judgments rather than reconstruct them from scratch each time.

This study also draws directly on the decisions-from-experience (DfE) literature, which shows that individuals learn from ongoing feedback and rely on past outcomes when making subsequent decisions (Erev & Haruvy, 2013; Erev & Ert, 2023; Hertwig & Erev, 2009). Unlike description-based choice models, the DfE framework explains how individuals construct preferences through experience, as memory retrieval, reinforcement, and sampling processes shape evaluative judgments over time (Erev & Ert, 2023; Pessach et al., 2025). This perspective is particularly relevant in OTA environments, where consumers repeatedly observe decision outcomes and adjust their evaluations accordingly.

Building on this foundation, we use Mittal et al.’s (1999) consumption system approach to explain how consumers assess and reweight subsystems of platform-mediated consumption, such as hotel choice attributes, online reviews, and service-related information, across repeated OTA usage. We also draw on Oliver’s (2010) satisfaction cycle model, rooted in expectancy disconfirmation theory, to clarify how and when satisfaction transforms into loyalty through temporal carryover effects.

The DfE literature complements these frameworks by specifying the behavioral mechanisms that drive reweighting and carryover. Specifically, it shows that individuals reinforce attributes that demonstrated predictive value in prior experiences and attenuate less diagnostic cues, thereby shifting evaluative weights instead of expanding criteria (Erev & Ert, 2023; Roth et al., 2016). This mechanism aligns closely with attribute reweighting in repeated platform use, where experiential feedback reorganizes the relative importance of existing evaluative criteria.

Together, these theories form an integrated framework. General living systems theory explains why evaluative judgments evolve. The consumption system approach clarifies how consumers recalibrate evaluative criteria across repeated decision episodes. The DfE perspective explains how experiential feedback and memory-based learning drive that recalibration (Erev & Haruvy, 2013; Erev & Ert, 2023; Hertwig & Erev, 2009). The satisfaction cycle model shows how cognitive continuity links prior and subsequent judgments through carryover effects. This integration allows us to capture the co-development of satisfaction and platform loyalty intentions over time as an expression of adaptive evaluative cognition within a dynamic OTA-mediated decision environment.

In platform-mediated decision environments, consumers do not evaluate service outcomes in isolation; they also assess the platform’s role in facilitating accurate and efficient choices. When platform-provided information, such as reviews, ratings, and recommendations, accurately predicts experienced service quality, consumers are more likely to attribute positive outcomes to the platform. This attribution process aligns with expectation confirmation and search efficiency perspectives, which explain how platforms build trust and loyalty by reducing uncertainty and enabling better choices.

**Figure 1:**
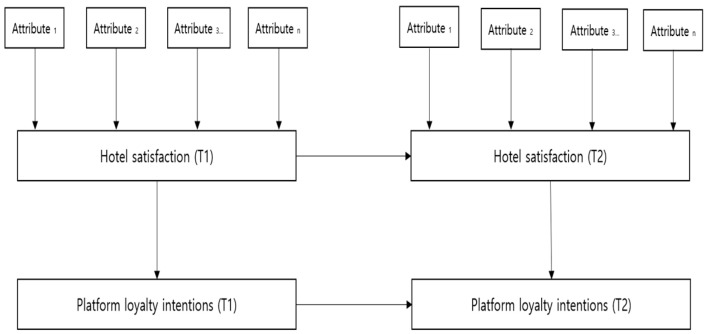
Proposed Research Model.

Our study’s proposed model (see Figure 1) incorporated three core elements of the consumption system: OTA-mediated hotel choice attributes, customer satisfaction with the hotel choice experience, and platform loyalty intentions. Each element has the capacity to evolve across subsequent consumption episodes (Bergkvist & Eisend, 2021).

Prior research has begun to recognize that the effects of online information vary over time. For example, Pan and Ha (2023) showed that online reviews exerted a stronger influence during the initial evaluation period but weakened in later periods. These findings suggest that attribute influence in platform-based environments shifts over time rather than remaining stable. From a behavioral science standpoint, consumers do not merely accumulate evaluative cues; instead, they selectively reinforce or attenuate them as experience shapes their decision confidence.

This pattern aligns with the DfE perspective, which posits that repeated experience leads individuals to rely more heavily on cues that previously yielded satisfactory outcomes, thereby strengthening those cues in subsequent decisions (Erev & Haruvy, 2013; Hertwig & Erev, 2009). Accordingly, we examine temporal change and carryover effects from an evolutionary perspective. Carryover effects occur when an evaluative state or intention at Time 1 (T1) influences the same construct at Time 2 (T2). Although Johnson et al. (2006) examined carryover effects on value, brand equity, and loyalty intentions in a mobile service renewal context, researchers have paid far less attention to how evaluative judgments persist and transfer during the shift from pre-consumption expectations to post-consumption experience in OTA-mediated hotel choice settings.

In this study, we treat OTA-mediated hotel choice attributes as time-varying predictors that shape satisfaction at each observation point (T1 and T2). Rather than modeling them as moderators or mediators, we position them as antecedents whose relative importance evolves as consumers move from search-based evaluation to experience-based judgment. This distinction matters because it frames attribute reweighting as an internal cognitive adjustment process rather than a contextual interaction effect.

From a decisions-from-experience perspective, this reweighting reflects experience-based learning. Individuals update the perceived diagnosticity of attributes based on past outcomes, which adaptively alters their influence on satisfaction judgments (Erev & Ert, 2023; Hertwig & Erev, 2009; Roth et al., 2016). This framework positions attribute evolution as the central mechanism through which platform-based learning reshapes the trajectory from satisfaction to platform loyalty intentions.

Service and electronic commerce research consistently shows that positive consumption experiences enhance customer satisfaction, which in turn fosters loyalty (Johnson et al., 2006; Loureiro & Kastenholz, 2011). Building on this insight, Oliver (1999) conceptualized loyalty as a progressive sequence that moves through cognitive, affective, and conative stages. From a behavioral science perspective, this framework is particularly relevant because it treats loyalty as an outcome of evolving evaluative judgments rather than mere behavioral repetition.

When we integrate this framework with DfE theory, we see that satisfaction reflects past evaluations and operates as a learned signal that guides future decisions. As individuals accumulate experience, they rely increasingly on prior evaluative outcomes when forming new judgments (Erev & Haruvy, 2013; Erev & Ert, 2023). In OTA contexts, this progression centers on the platform, as consumers repeatedly use the same OTA to search, evaluate, and book hotels. Accordingly, we extend Oliver’s (1999) loyalty framework by examining how changes in the salience of hotel choice attributes across repeated OTA-mediated episodes affect platform loyalty intentions. Specifically, we identify which attribute shifts reinforce or weaken the satisfaction–platform loyalty link, thereby providing a process-oriented account of how evaluative cognition stabilizes or intensifies over time.

Our conceptual model outlines a sequential process that links OTA-mediated hotel choice experiences to satisfaction and, ultimately, to platform loyalty intentions. Since platform loyalty intentions evolve across consumption episodes (Johnson et al., 2006), we must understand consumers’ cumulative OTA-facilitated hotel experiences to capture satisfaction accurately. Within this framework, consumers’ perceptions of platform-presented and platform-filtered hotel choice attributes directly influence satisfaction. Consistent with Oliver’s (2010) satisfaction cycle model, satisfaction then drives platform loyalty intentions.

From a behavioral decision-making perspective, this process reflects memory-based continuity: individuals carry forward prior evaluative outcomes and integrate them into subsequent judgments, consistent with experience-based learning mechanisms (Erev & Ert, 2023; Hertwig & Erev, 2009). Therefore, we conceptualize satisfaction as a dynamic evaluative judgment that integrates prior experiences through cognitive carryover. By embedding this process in a longitudinal framework, we position satisfaction as a dynamic mediator that translates evolving evaluative criteria into continued decision commitment.

Consumer behavior theory further posits a sequential cause-and-effect relationship linking satisfaction and loyalty over time. Specifically, satisfaction formed during an initial OTA-mediated booking experience (T1) influences platform loyalty intentions at T1, which then shape subsequent booking behaviors and evaluative standards at T2. These subsequent experiences affect satisfaction at T2, and ultimately reinforce or weaken platform loyalty intentions. Although Figure 1 does not explicitly depict the direct path from T1 platform loyalty intentions to subsequent booking behavior, Mittal et al.’s (1999) consumption system framework supports this linkage by explaining how loyalty formed in earlier episodes shapes evaluative criteria in later encounters.

This temporal interdependence aligns with experience-based decision models, which show that reinforcement and learning from past outcomes guide repeated choices rather than independent evaluation at each decision point (Erev & Ert, 2023; Hertwig & Erev, 2009). This temporal interdependence highlights how learning, memory, and judgment reinforcement drive experience-based decision-making and underscores the iterative, learning-based nature of platform loyalty formation in OTA-mediated hotel choice.

Beyond the consumption system framework and satisfaction cycle model, we also situate this study within a broader literature on the dynamic nature of satisfaction and loyalty in service and platform contexts. Prior studies demonstrate that satisfaction and loyalty evolve as consumers accumulate experience, form expectations, and update their evaluations (Johnson et al., 2006; Mittal et al., 1999; Oliver, 1999). Studies on customer retention further show that prior satisfaction and platform loyalty intentions carry over into future evaluations and behaviors, revealing the temporal interdependence of consumer judgments (Johnson et al., 2006). Building on this literature, we extend the dynamic perspective by examining how satisfaction persists over time and functions as a cognitive anchor that shapes subsequent evaluations and decision processes.

From a social psychological perspective, this study connects to research on memory, learning, and experience-based evaluation in shaping consumer judgments (Erev & Ert, 2023; Hertwig & Erev, 2009). Prior work shows that accumulated experience increases the accessibility and stability of evaluative judgments, which leads individuals to rely more heavily on prior evaluations in later decisions. In this regard, conceptualizing satisfaction as a cognitive anchor aligns with theories of attitude strength and memory-based evaluation, which posit that prior judgments can serve as stable reference points for future evaluations. By incorporating this perspective, we offer a behavioral interpretation of how evaluative judgments become progressively anchored over time.

Finally, this study contributes to the literature on platform-mediated decision-making by highlighting how intermediaries shape evaluative processes. Prior research shows that platforms facilitate information search, reduce uncertainty, and support decisions by providing reviews, ratings, and recommendations (Albayrak & Caber, 2015; Qu et al., 2000). Extending this line of research, the present study demonstrates that ongoing platform interactions influence decision outcomes and the cognitive processes underlying evaluation. By examining how satisfaction and attribute evaluations evolve within an OTA context, we clarify how platform-mediated experiences shape the temporal dynamics of evaluative cognition.

## 3. Hypothesis Development

### 3.1. Relationship Between Hotel Choice Attributes and Satisfaction over Time

Hotel attributes serve as critical determinants in consumers’ accommodation decision-making processes. From a behavioral science perspective, consumers use these attributes as evaluative criteria to construct and update judgments as they move from pre-consumption expectations to post-consumption experiences. In OTA contexts, consumers primarily encounter, compare, and evaluate these attributes within platform-mediated information environments. Although hospitality, tourism, and service scholars have examined various hotel attributes, we focus on empirical studies published between 2010 and 2020, a decade marked by significant growth in digitally mediated hotel search and booking behavior. During this period, researchers identified more than 60 distinct hotel attributes associated with customer satisfaction and choice behavior (Albayrak & Caber, 2015; Bodet et al., 2017; Caber & Albayrak, 2014; Chou & Chen, 2014; Francesco & Roberta, 2019; Jang et al., 2018; J. Kim et al., 2020).

For analytical clarity and empirical relevance, this study focuses on 15 attributes most frequently examined in prior research: OTA-mediated perceptions of hotel room quality, safety and security, food and beverage offerings, leisure activity availability, room pricing, reputation, staff politeness, cleanliness, relaxing lounge or bar availability, parking facilities, information provision about the surrounding area, luggage service, concierge service, swimming pool availability, and visitor reviews. Together, these attributes represent the multidimensional cues through which consumers evaluate hotels within OTA platforms and provide a robust foundation for examining how they form and revise evaluative judgments. Rather than treating hotel quality as a single aggregate control variable, we operationalize it through multiple evaluative attributes (e.g., perceived hotel room quality, perceived room price, and online reviews), reflecting the multidimensional nature of consumer evaluation.

The inclusion of these 15 attributes is theoretically grounded in the consumption–system framework (Mittal et al., 1999) and service quality theory, which conceptualize satisfaction as arising from both functional and experiential subsystems within the consumption experience. Accordingly, the selected attributes capture core functional dimensions (e.g., room quality, cleanliness, safety, pricing) and experiential dimensions (e.g., food and beverages, leisure facilities, concierge services, visitor reviews) that jointly determine satisfaction formation in platform-mediated hotel choice contexts. This multidimensional configuration aligns with adaptation-level theory (Helson, 1964), which suggests that consumers continuously recalibrate attribute importance based on accumulated experience. From a behavioral standpoint, this recalibration reflects an adaptive learning process through which evaluative criteria are selectively reweighted as experience reshapes reference standards. Such recalibration is particularly salient in OTA environments, where repeated exposure to platform information and post-consumption feedback accelerates learning.

To ensure theoretical rigor, the fifteen hotel choice attributes were categorized into two higher-order dimensions: tangible (functional) and intangible (experiential), based on Zeithaml (1988), Mittal et al. (1999), and Ding and Keh (2017). Tangible attributes include hotel room quality, cleanliness, safety and security, perceived room price, parking facilities, swimming pool availability, and food and beverage offerings, and represent performance-based features that consumers can evaluate during OTA-based search and post-consumption assessment. In contrast, intangible attributes include staff politeness, hotel reputation, concierge services, luggage services, leisure activities, relaxing lounge or bar availability, information provision about the surrounding area, and visitor reviews, which rely on experiential and affective evaluations shaped through service interaction and accumulated experience. This two-dimensional classification reflects the distinction between search-based and experience-based evaluations. According to construal level theory (Ding & Keh, 2017), tangible attributes dominate initial evaluations, while intangible attributes gain prominence as psychological distance decreases through repeated experiences—a pattern consistent with experience-driven shifts in evaluative cognition rather than changes in available information.

Given that hotel attribute evaluations are shaped by consumers’ service experiences, researchers have shown sustained interest in understanding how specific attributes contribute to overall satisfaction (Albayrak & Caber, 2015; Srivastava & Kumar, 2021). However, most existing studies employ importance–performance analysis (IPA) without fully defining satisfaction as the aggregate outcome of individual-level attribute evaluations. To address this limitation, this study adopts Mittal et al.’s (1999) consumption–system approach and Oliver’s (1993) expectancy–disconfirmation theory, conceptualizing satisfaction as a function of performance-based individual-level evaluations of OTA-mediated hotel choice attributes. This perspective treats satisfaction as an integrative evaluative judgment rather than a momentary affective response, enabling closer examination of how judgment standards evolve over repeated decisions.

Defining satisfaction as a function of individual-level attribute evaluations offers two theoretical advantages. First, it reflects consumers’ memory-based perceptions of past OTA-mediated booking and consumption experiences, which shape subsequent satisfaction judgments. Second, it enables systematic analysis of temporal shifts in attribute salience, thus allowing researchers to identify which attributes gain or lose importance across repeated consumption episodes (J. M. Kim et al., 2023). Such shifts provide direct insight into how evaluative criteria are reorganized as decision confidence and experiential knowledge accumulate.

Although most prior studies have adopted a cross-sectional approach to attribute evaluation, a longitudinal perspective better captures the dynamic evolution of attribute weights. For example, Zhang and Ha (2023) found that food and beverage pricing was a salient satisfaction determinant during initial visits (T1), but its relative importance decreased during subsequent visits (T2). Conversely, attributes such as cultural or contextual experiences gained prominence in later consumption episodes. These findings underscore the need to move beyond static models and adopt a dynamic analytical framework capable of capturing experience-driven reweighting of evaluative criteria in OTA-mediated hotel choice.

Recognizing that consumers assign varying levels of importance to individual service attributes, this study posits that changes in these weights are central to understanding how satisfaction evolves. As experience accumulates, evaluations may shift toward attributes that offer greater perceived value or emotional resonance. Concurring with LaTour and Peat (1979), satisfaction is viewed as a cumulative evaluative judgment constructed from attribute-level assessments. As such, changes in attribute weights are interpreted as indicators of adaptive evaluative learning rather than as random fluctuations in preference. Thus, the following hypothesis is proposed.

**H1.**  *The relative weights of evaluative criteria evolve over time as individuals accumulate experience through OTA-mediated decisions.*

### 3.2. Satisfaction–Platform Loyalty Intentions Relationship over Time

This study defines satisfaction as a cognitive–affective evaluation that arises when consumers compare perceived hotel performance with prior expectations (Oliver, 1993, 2010). This definition builds on the expectancy–disconfirmation paradigm, which explains satisfaction as the result of confirming or disconfirming prior expectations. From a behavioral science perspective, we further conceptualize satisfaction as an evaluative judgment that integrates prior experiences and serves as a reference point for subsequent decisions. Accordingly, we treat satisfaction as a cumulative and dynamic process that evolves as consumers gain OTA-mediated hotel choice experience, recalibrate expectations, and refine evaluative baselines.

Platform loyalty intentions refer to a consumer’s predisposition to repeatedly use the same online travel agency for future hotel bookings and to recommend the platform to others. Rather than viewing loyalty intentions solely as behavioral repetition, this study interprets them as expressions of increasing cognitive commitment and reliance in repeated decision-making. Prior research has demonstrated that the satisfaction–loyalty linkage strengthens as consumers gain repeated positive experiences with a service provider (Johnson et al., 2006; Mittal et al., 1999; Oliver, 2010). In platform contexts, this iterative reinforcement process reflects growing reliance on the platform as the primary interface for search, evaluation, and transaction, consistent with Oliver’s satisfaction cycle theory.

However, empirical evidence suggests that this relationship may not always progress linearly. Mittal et al. (1999) found that in certain industries, satisfaction–loyalty linkages weaken as usage experience increases due to shifting attribute weights and elevated expectations. Similarly, Kumar et al. (2013) argued that the satisfaction–loyalty relationship is not static but may fluctuate across different consumption process stages. These findings highlight the possibility of nonlinear or asymmetric trajectories, including satisfaction plateaus, diminishing marginal effects, or temporary declines when expectations increase faster than performance. From a behavioral standpoint, these patterns suggest that satisfaction may change its functional role in decision-making as evaluative criteria and reference standards evolve. Building on this insight, the present framework conceptualizes the satisfaction–platform loyalty relationship as inherently adaptive rather than uniformly progressive. This perspective emphasizes that the influence of satisfaction depends on how it is cognitively interpreted and reinforced over time, rather than on its absolute level at a single point.

In OTA environments, consumers are continuously exposed to competing alternatives, making prior satisfaction a critical reinforcing mechanism that fosters continued platform usage. Guided by adaptation-level theory (Helson, 1964), we posit that as consumers accumulate positive OTA-mediated hotel choice experiences, their reference levels adjust upward, leading satisfaction to function increasingly as a signal of decision confidence and evaluative certainty rather than mere post-consumption affect. As a result, satisfaction is expected to exert a progressively stronger influence on subsequent platform-related decisions as experience accumulates. Thus, the following hypotheses are proposed:

**H2.**  *Satisfaction at T1 has a direct and positive effect on platform loyalty intentions at T1, reflecting its role as an initial evaluative judgment in ongoing decision-making.*

**H3.**  *The effect of satisfaction on platform loyalty intentions strengthens over time (T2) as evaluative criteria stabilize and decision confidence increases through experience-driven reweighting.*

### 3.3. Carryover Effects of Satisfaction and Platform Loyalty Intentions

The present study highlights the role of carryover effects, that is, the influence of prior psychological states on subsequent evaluations, in explaining how satisfaction and platform loyalty intentions evolve. From a behavioral science perspective, carryover effects reflect the persistence of evaluative cognition: prior judgments shape later evaluations through memory and learning rather than independent reassessment. Tourangeau and Rasinski (1988) explain that carryover effects occur when responses at one time point systematically influence evaluations at a later time point. Drawing on Oliver’s (2010) satisfaction cycle model and Johnson et al.’s (2006) loyalty evolution framework, we posit that satisfaction and platform loyalty intentions at T1 serve as foundational inputs that shape satisfaction and loyalty at T2 by anchoring evaluative reference points across decision episodes.

From this perspective, consumer behavior—such as repeatedly booking hotels through the same OTA—is not driven by isolated service encounters but reflects a cumulative psychological process in which prior satisfaction increases future satisfaction. This pattern reflects cognitive continuity, whereby earlier evaluative judgments are carried forward and integrated into subsequent evaluations rather than being discarded. This continuity reinforces platform loyalty intentions, which are likewise shaped by earlier dispositions. Specifically, satisfaction at T2 is conceptualized as a function of satisfaction at T1, and platform loyalty intentions at T2 are partially determined by platform loyalty intentions at T1 (Pee et al., 2019). Together, these relationships illustrate a recursive evaluative process in which judgments and decision commitments are temporally interdependent. These propositions underscore the dynamic and recursive nature of platform-based consumption, wherein evaluations and intentions are temporally interconnected rather than independent. Importantly, this interdependence suggests that repeated decisions are structured by memory-based judgment reinforcement rather than by repeated formation of new evaluative standards. Thus, the following hypotheses are proposed:

**H4.**  *Prior satisfaction at T1 positively affects satisfaction at T2, reflecting the persistence of evaluative judgments through cognitive carryover.*

**H5.**  *Prior platform loyalty intentions at T1 positively affect platform loyalty intentions at T2, indicating continuity in decision commitment between the pre-consumption and post-consumption phases.*

## 4. Methodology

### 4.1. Data Collection

To empirically test the proposed hypotheses, this study employed a two-wave longitudinal panel design in collaboration with a professional Korean online research firm. A two-time-lag panel design is particularly suitable for behavioral science research examining how evaluative judgments evolve across repeated decision episodes. Structured surveys were distributed to individuals who had made a hotel reservation through an online travel agency for an upcoming summer vacation at a 4- or 5-star hotel. To ensure sample alignment with the study’s objectives and enhance external validity, participants were required to: (1) have completed a hotel booking via an OTA for an upcoming stay at a 4- or 5-star hotel at the time of the first survey (T1), and (2) have not yet stayed at the booked hotel at T1.

This panel-based approach enabled the collection of pre-consumption evaluations formed during OTA-based search and booking (T1) and post-consumption evaluations following the actual stay (T2) from the same participants, thereby capturing the temporal evolution of consumer perceptions within an OTA-mediated hotel choice process. By observing the same individuals before and after direct experience, the design allows examination of how evaluative criteria and judgments are updated through experience rather than inferred from cross-sectional differences. According to the Korea Tourism Organization’s classification, 4-star hotels offer comprehensive facilities such as multiple restaurants, business centers, and fitness facilities, and represent relatively standardized service levels. Restricting the sample to 4- or 5-star hotels helped control for extreme service heterogeneity, thereby isolating cognitive changes in evaluation from confounding variations in baseline service quality, allowing the analysis to focus on changes in attribute evaluation, satisfaction, and platform loyalty intentions rather than basic quality differences.

The first wave (T1) was conducted in July 2025, shortly after participants completed their OTA-based hotel bookings but before their actual stays. The second wave (T2) occurred three months later, after participants had completed their stays. This time lag was designed to capture post-experience evaluative judgments after sufficient temporal distance for reflection and memory consolidation. Only those respondents who confirmed that they had stayed at the same hotel booked via the OTA at T1 were retained for T2, ensuring a direct linkage between OTA-based expectations and post-consumption evaluations. Invitations were sent to 1257 screened participants, yielding 751 T1 responses. After excluding careless responses based on instructed-response checks and consistency indices (Meade & Craig, 2012), 721 valid responses were retained.

At T2, the survey was administered to the same respondents using identical procedures, with 538 participants completing it. After applying the same data quality filters, 481 valid paired responses remained, representing a 38.2% panel retention rate. To minimize attrition, those completing both waves received a small incentive (a Starbucks voucher worth approximately USD 3.26). This retention rate is consistent with longitudinal survey research involving behavioral evaluations across multiple time points.

The final sample (n = 481) consisted of 46% male respondents, with a mean age of 33.2 years. Most participants (68.2%) were employed in office-based occupations, with an average annual income of USD 42,600, reflecting a stable middle-income demographic. On average, participants stayed 2.8 nights at the hotel booked at T1, and all reported that the stay at T2 was their first at that property, ensuring that satisfaction and platform loyalty evaluations reflected newly formed OTA-mediated experiences rather than prior familiarity. This condition strengthens internal validity by ensuring that observed evaluative changes reflect learning from the focal decision episode rather than accumulated brand-specific familiarity.

### 4.2. Measures

Table 1 presents the survey measures used to test the proposed model. We assessed satisfaction with the hotel choice experience using three items adapted from Gallarza and Saura (2006) and El-Adly (2019), and we refined the wording to emphasize an expectancy–disconfirmation-based evaluation within an OTA-mediated booking and consumption process. Consistent with a behavioral science perspective, we treat satisfaction as an integrative evaluative judgment through which individuals cognitively reconcile expectations with experienced outcomes.

We measured platform loyalty intentions using three items adapted from Johnson et al. (2006) and El-Adly (2019), modifying them to capture respondents’ intentions to reuse the same OTA for future hotel bookings and to recommend the platform to others. We operationalize loyalty as platform loyalty intentions rather than as observed behavior. In line with the study’s conceptual framework, we interpret these intentions as indicators of decision commitment and cognitive reliance rather than mere behavioral repetition.

To ensure comparability over time, we used identical measurement items for all multi-item constructs at T1 and T2. We kept the wording unchanged across waves, which allows direct comparison of construct meanings over time. This approach supports measurement equivalence and ensures that any observed differences in satisfaction and platform loyalty intentions reflect changes in evaluative judgments.

Furthermore, the study evaluated the following 15 OTA-mediated hotel choice attributes: hotel room quality, safety and security, food and beverage offerings, leisure activities, perceived room price, hotel reputation, staff politeness, cleanliness, relaxing lounge or bar availability, parking facilities, information provided about the surrounding area, luggage services, concierge services, swimming pool availability, and hotel visitor reviews. These attributes are conceptualized as evaluative cues that individuals use to construct judgments during repeated decision-making.

All hotel choice attributes were measured using single-item indicators. While multi-item measures are generally preferred, prior studies have shown that under certain conditions—particularly when assessing concrete, specific, and easily identifiable attributes—single-item measures can perform comparably to multi-item scales (Allen et al., 2022; Diamantopoulos et al., 2012). From a behavioral science standpoint, single-item indicators are appropriate when the goal is to capture salient cognitive representations of distinct evaluative criteria rather than latent trait dimensions. Given that the attributes examined in this study represent distinct and clearly defined cues commonly encountered on OTA platforms, single-item measures are appropriate for capturing attribute-level evaluations within a dynamic consumption system.

The selection of these 15 attributes was informed by existing hospitality and service research (Albayrak & Caber, 2015; Bodet et al., 2017; Caber & Albayrak, 2014; J. Kim et al., 2019; Law & Hsu, 2005; Qu et al., 2000; Srivastava & Kumar, 2021) and reflects attributes that are prominently displayed, compared, and evaluated in OTA environments. Their prominence ensures that respondents can readily access stable mental representations of these criteria when reporting evaluations. All measures were rated on a 5-point Likert scale ranging from 1 (strongly disagree/not very important) to 5 (strongly agree/very important). A detailed list of all attribute measurement statements and their sources is provided in Appendix A.

### 4.3. Common Method Bias

Given the well-documented strong correlation between satisfaction and platform loyalty intentions in the existing service and electronic commerce literature, we assessed the possibility of common method bias to ensure the robustness of our findings. This assessment is particularly important in behavioral science research examining evaluative judgments over time, as observed continuity effects may otherwise be attributed to measurement artifacts rather than genuine cognitive persistence. In line with variance-based SEM practices, we adopted a PLS-SEM-consistent diagnostic approach rather than relying on covariance-based model comparison techniques.

Specifically, common method bias was evaluated using the full collinearity variance inflation factor (VIF) approach (Kock, 2015), which has been increasingly recommended as an effective diagnostic tool for detecting common method variance in PLS-SEM models. This approach examines whether any latent construct—such as satisfaction or platform loyalty intentions—exhibits excessive collinearity with other constructs in the model. By applying this procedure, we aimed to ensure that the observed relationships reflect substantive evaluative processes rather than shared method-induced variance.

All inner variance inflation factor (VIF) values were below 3.0, well under the conservative threshold of 3.3. This result suggests that neither multicollinearity nor common method bias poses a serious concern in this study. Therefore, the observed relationships between satisfaction and platform loyalty intentions likely reflect substantive associations. Accordingly, we interpret the temporal strengthening and carryover effects identified in the longitudinal model as genuine cognitive and evaluative dynamics.

Although the full collinearity VIF test provides a useful statistical diagnostic, we acknowledge the limitations of relying on a single test. Therefore, we implemented several procedural remedies to mitigate potential common method bias. Specifically, we adopted a longitudinal design with temporal separation between T1 and T2, which reduces respondents’ ability to rely on prior responses when answering subsequent questions. We also differentiated the substantive focus of the two waves: T1 captures pre-consumption expectations, and T2 captures post-consumption evaluations. Together, these design features reduce the likelihood that common method bias affected the results. Overall, the proposed model demonstrates robust support within an experience-based longitudinal framework.

### 4.4. Measurement Comparison

Shifts in satisfaction, platform loyalty intentions, and OTA-mediated hotel choice attributes were compared across the two measurement periods. This comparison provides an initial descriptive assessment of whether evaluative judgments exhibit systematic change as individuals move from pre-consumption expectations to post-consumption experience. As shown in Table 2, a pairwise *t*-test was conducted to determine whether the differences in the means of the constructs and attributes between T1 and T2 were statistically significant.

The results indicate that mean-level changes across the two periods were significant, suggesting that participants’ OTA-mediated consumption evaluations evolved over time as experience accumulated. From a behavioral science perspective, these mean-level shifts reflect changes in evaluative reference points rather than random fluctuation, consistent with experience-driven judgment updating. Notably, the Cronbach’s alpha values for both satisfaction and platform loyalty intentions remained stable across the two periods, indicating consistent internal reliability despite temporal changes in construct levels. This stability suggests that the underlying evaluative constructs remained conceptually coherent over time, even as their expression and intensity changed through experience.

### 4.5. Non-Response Bias

Non-response bias was assessed using the extrapolation method. Assessing non-response bias is particularly important in longitudinal behavioral research, as systematic attrition could otherwise be confounded with apparent changes in evaluative judgments over time. Specifically, participants who completed the initial survey (T1) but did not participate in the second wave (T2) were compared with those who completed both surveys.

Following Armstrong and Overton’s (1977) extrapolation approach, independent-samples *t*-tests were conducted to compare mean differences in key constructs (satisfaction and platform loyalty intentions) and demographic characteristics. The results revealed no statistically significant differences at *p* < 0.05, indicating that non-response bias is unlikely to be a concern in the present study. This finding suggests that the observed temporal changes in satisfaction and platform loyalty intentions are unlikely to be driven by selective panel attrition and can therefore be interpreted as reflecting genuine evaluative and decision-related dynamics.

### 4.6. Control Variables

We included two control variables: hotel size and hotel location. These variables capture contextual features of the consumption environment that may influence evaluative judgments independently of platform-mediated decision processes. These variables may be significantly related to participants’ perceptions of OTA-mediated hotel choice attributes, satisfaction, and platform loyalty intentions. To this end, participants indicated hotel size as either (1) medium or (2) large. Hotel location was categorized into four groups: (1) metropolitan, (2) coastal, (3) mountainous, and (4) island areas.

These control variables were incorporated into the proposed model to reduce unobserved heterogeneity associated with contextual characteristics of the hotel stay, thereby isolating changes in evaluative cognition from variation driven by structural or environmental hotel characteristics. By accounting for these contextual influences, the analysis remains centered on experience-driven changes in evaluative criteria and decision commitment rather than on differences attributable to hotel scale or geographic setting.

### 4.7. Attrition Analysis

Given the final panel retention rate of 38.2%, we conducted an attrition analysis to examine whether participants who completed both waves (retained group) differed systematically from those who completed only T1 (dropped group). Researchers must conduct this step in longitudinal behavioral research because selective attrition could lead them to misinterpret sample differences as temporal change in evaluative judgments.

We based the analysis on T1 data, including demographic variables (gender, age, occupation, and income), key study constructs (T1 satisfaction and platform loyalty intentions), and key hotel choice attributes that exhibited substantial changes across time (e.g., visitor reviews, hotel room quality, and perceived room price). Table 2 summarizes the respondents’ demographics.

Independent-samples *t*-tests and χ^2^ tests revealed no statistically significant differences between the retained and dropped groups across these variables (all *p* > 0.10). The magnitude of the differences was negligible, indicating high comparability at T1. Importantly, the two groups did not differ in initial satisfaction or platform loyalty intentions, which suggests that respondents with less favorable initial evaluations were not more likely to drop out between T1 and T2.

These findings indicate that attrition occurred randomly rather than systematically, and that the final longitudinal sample does not suffer from selective dropout bias. Therefore, the observed changes in satisfaction, attribute weighting, and platform loyalty intentions over time likely reflect within-individual evaluative updating rather than sample selection effects. Table 3 reports the detailed results. Consistent with these findings, the small differences between retained and dropped groups further suggest that attrition bias does not meaningfully affect the results.

### 4.8. Data Analysis Method

We implemented partial least squares structural equation modeling (PLS-SEM) in SmartPLS to test the proposed structural model. PLS-SEM allows us to examine how relationships among evaluative judgments evolve rather than merely test static equilibrium models. It is particularly well-suited for predictive research involving complex relationships among latent constructs, especially in electronic commerce and platform-based consumer behavior contexts (Chin et al., 2008). Compared with covariance-based SEM, PLS-SEM provides greater robustness when estimating models with relatively complex structures, moderate sample sizes, and non-normal data distributions (Anderson & Swaminathan, 2011).

In addition, our model combines multi-item reflective constructs with single-item indicators for specific attributes, and PLS-SEM accommodates this structure effectively. Given the study’s predictive orientation and the model’s complexity relative to the sample size, we selected PLS-SEM because it provides a robust estimation approach that is less sensitive to distributional assumptions (Chin et al., 2008; Hair et al., 2013).

Furthermore, PLS-SEM accommodates formative specifications and single-item indicators, making it particularly appropriate for models that include attribute-level evaluations and dynamic carryover effects across time (Hair et al., 2013). This flexibility allows the analysis to capture changes in the relative influence of evaluative criteria without imposing restrictive assumptions about measurement invariance across decision episodes. Given the study’s longitudinal design and its focus on evolving relationships among hotel choice attributes, satisfaction, and platform loyalty intentions, PLS-SEM offers a methodologically appropriate and theoretically consistent analytical approach for modeling experience-driven changes in evaluative cognition and decision commitment.

## 5. Results

Before testing the proposed structural model, we evaluated the measurement model using PLS-SEM to assess construct reliability and validity. During this stage, we removed one item from the platform loyalty intentions construct (“I would continue using this OTA in the future even if prices increased”) because its standardized loading was below 0.70, failing to meet the recommended threshold for convergent validity (Fornell & Larcker, 1981; Hair et al., 2013). To maintain measurement equivalence across time, we excluded this item in T1 and T2. Following Netemeyer et al. (2003), we eliminated low-loading items to enhance construct reliability and parsimony.

As Table 4 shows, all outer loadings ranged from 0.74 to 0.89, exceeding the recommended minimum of 0.70. Composite reliability and Cronbach’s alpha values for each construct, including satisfaction and platform loyalty intentions, were also above the 0.70 cutoff. Furthermore, the average variance extracted (AVE) for all constructs exceeded the recommended threshold of 0.50 (Hair et al., 2013). The measurement model demonstrated satisfactory convergent validity at both T1 and T2. To assess longitudinal comparability, we compared factor loadings across the two time points. All items exhibited consistently high loadings across both waves, with only minor variations in magnitude. This pattern demonstrates stable and comparable measurement over time and supports longitudinal measurement stability.

To assess discriminant validity, two complementary approaches were employed. First, Fornell and Larcker’s (1981) criterion was applied, which requires that the square root of each construct’s AVE exceeds its correlations with other constructs. As presented in Table 4, both satisfaction and platform loyalty intentions satisfied this criterion, providing evidence of discriminant validity.

Second, in response to critiques of Fornell and Larcker’s (1981) approach, the Heterotrait–Monotrait Ratio (HTMT) method was also utilized. From a conservative perspective, HTMT values above 0.85 may indicate potential discriminant validity concerns (Henseler et al., 2015). As reported in Table 1, all HTMT values were below this threshold (<0.85), further confirming that discriminant validity was achieved for all constructs in the model.

### Model Estimates

We present the results in the following order. First, we evaluate the model’s explanatory and predictive power. Next, we report attribute-level findings from hypothesis testing. This structure allows us to assess the overall stability of evaluative relationships and the specific mechanisms through which judgments evolve during the transition from pre-consumption expectations to post-consumption experience.

**Figure 2:**
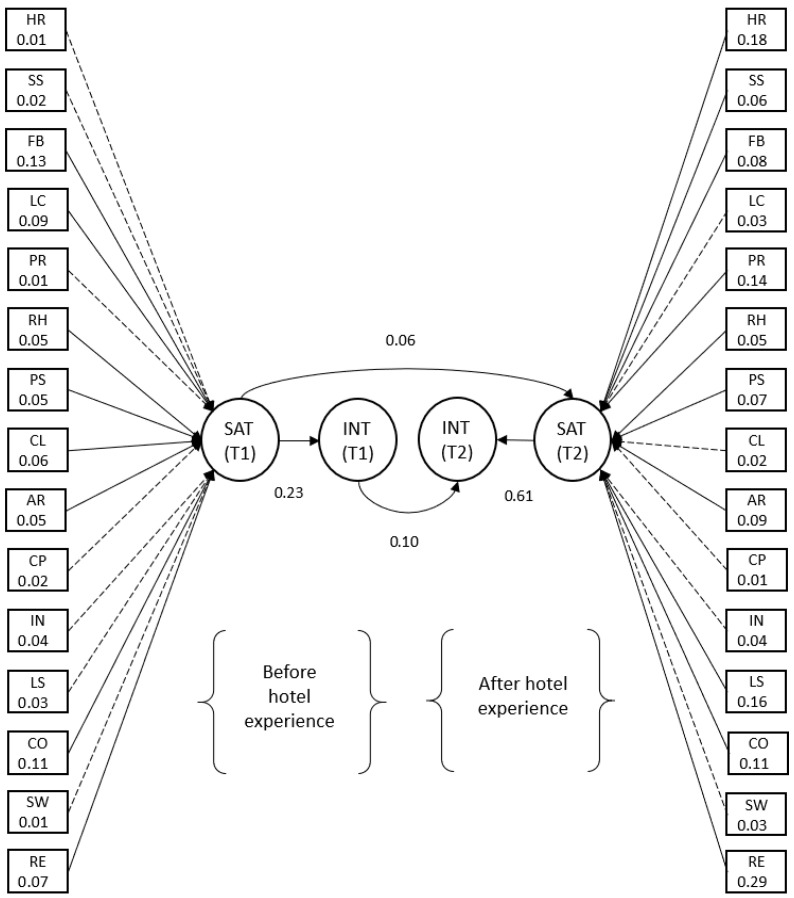
Path estimates. Notes: The direct lines = significant; The dashed lines = not significant. The dotted line is not significant at *p* < 0.05. SAT = satisfaction; INT = platform loyalty intentions; HR = hotel room quality; SS = safety and security; FB = food and beverage; LC = leisure activities; PR = perceived room price; RH = hotel reputation; PS = level of staff politeness; CL = cleanliness; AR = relaxing lounge or bar availability; CP = parking; IN = various information provided around hotel; LS = luggage service; CO = concierge service; SW = swimming pool; RE = hotel visitor reviews.

We examined the R^2^ values of the four endogenous constructs to evaluate overall explanatory power. As Figure 2 illustrates, all R^2^ values reached satisfactory levels (satisfaction T1: R^2^ = 0.20; platform loyalty intentions T1: R^2^ = 0.31; satisfaction T2: R^2^ = 0.57; platform loyalty intentions T2: R^2^ = 0.39). Notably, the substantially higher R^2^ values at T2 indicate that post-experience evaluations are more structurally organized and predictable, consistent with the stabilization of evaluative cognition through experience.

To establish predictive significance in PLS-SEM, we also assessed predictive relevance using the Q^2^ statistic. Exogenous variables play a critical role in determining the predictive relevance of endogenous constructs, and a Q^2^ value greater than zero indicates adequate predictive relevance (Hair et al., 2013). In this study, satisfaction (T2) achieved a Q^2^ of 0.51, and platform loyalty intentions (T2) achieved a Q^2^ of 0.37. These values confirm strong predictive relevance and suggest that post-experience judgments are stronger and more systematically structured than pre-experience evaluations.

The results for H1 reflect shifts in the weights of individual hotel choice attributes. More than half of the attributes became more salient in shaping satisfaction over time, whereas three attributes—hotel reputation, provision of local information, and concierge services—remained relatively stable (see Table 5).

Notably, the weights of hotel room quality (Δ = 0.17) and visitor reviews (Δ = 0.22) increased significantly, suggesting that these attributes gained importance after consumers accumulated OTA-mediated hotel choice experience. The partial stability of several attributes further suggests that attribute reweighting operates selectively rather than universally. Experience reorganizes evaluative hierarchies by reinforcing a subset of criteria instead of introducing new ones. Therefore, we find partial support for H1.

H2 tested the extent to which satisfaction positively affected platform loyalty intentions at T1, as determined by T1 evaluations of OTA-mediated hotel choice attributes. The results reveal that the proposed relationship was positive and statistically significant (β = 0.23, *p* < 0.01), supporting H2. This finding indicates that even at the pre-consumption stage, satisfaction functions as an initial evaluative signal that informs subsequent decision commitment.

H3 focused on how the satisfaction–platform loyalty intentions relationship evolved over time. As illustrated in Figure 2, this relationship strengthened as consumers accumulated OTA-mediated consumption experience (T1: β = 0.23, *p* < 0.01 vs. T2: β = 0.61, *p* < 0.01). This finding indicates that satisfaction plays an increasingly important role in shaping continued platform usage intentions as experience accumulates. This pattern suggests that satisfaction increasingly serves as a source of decision confidence and cognitive reliance in repeated decision-making. Notably, this result contrasts with Mittal et al.’s (1999) finding that the satisfaction–loyalty relationship weakens over time, as proposed by the consumption-system approach.

Directly comparing the magnitudes of the path coefficients at T1 and T2 is insufficient to conclude that the satisfaction–platform loyalty intentions relationship has evolved, as such differences may reflect sampling variability rather than true structural change. Accordingly, we conducted a permutation-based multi-group analysis within the PLS-SEM framework to test whether the satisfaction–platform loyalty intentions path differed across time.

We treated T1 and T2 as separate groups, and evaluated the null hypothesis of equal path coefficients (β_T1 = β_T2) using 5000 random permutations. The results indicate that the T2 path coefficient (β = 0.61) significantly exceeds the T1 coefficient (β = 0.23), yielding a difference of Δβ = 0.38 (*p* = 0.019). These results provide direct evidence that the strengthening of the satisfaction–platform loyalty relationship reflects a genuine temporal shift in evaluative structure rather than random fluctuation.

To address potential concerns about the paired nature of the data, we conducted an additional within-subject analysis using a difference-score approach. This approach explicitly models within-individual change by comparing each respondent’s T2 and T1 evaluations. We computed changes in satisfaction and platform loyalty intentions using latent variable scores and then examined the relationship between these changes.

As Table 6 shows, changes in satisfaction (ΔSAT) positively and significantly predict changes in platform loyalty intentions (ΔLOY) (β = 0.26, *p* < 0.001), indicating that increases in satisfaction correspond to increases in loyalty over time. To determine whether attribute reweighting reflects within-individual evaluative updating, we also analyzed key attributes that exhibited substantial temporal changes. The results show that changes in core attributes—particularly hotel room quality and visitor reviews—significantly predict changes in satisfaction, whereas other attributes exert comparatively weaker effects.

Overall, these findings confirm that the temporal strengthening of the satisfaction platform loyalty relationship and the selective reinforcement of key evaluative criteria remain robust when accounting for within-individual dependence. The results do not reflect methodological artifacts associated with the independent group assumption. We further assessed robustness by examining bootstrapped confidence intervals for the key path coefficients; none of the intervals included zero for the main effects, which provides additional support for the statistical significance of the results.

H4 and H5 examined the carryover effects of satisfaction and platform loyalty intentions across time. The analysis revealed that the standardized path coefficients for the carryover effects of satisfaction and platform loyalty intentions were 0.06 and 0.10, respectively (*p* < 0.05).

**Figure 3:**
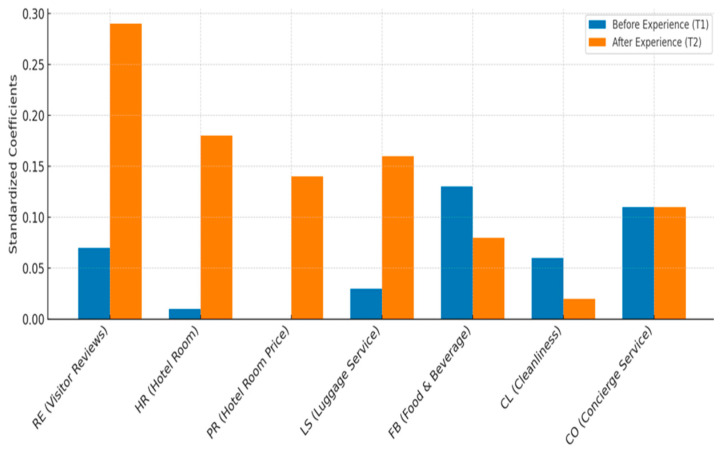
Attribute Influence on Satisfaction (T1 vs. T2).

These findings are consistent with Oliver’s (2010) satisfaction cycle framework and provide empirical support for both H4 and H5. Although modest in magnitude, these effects indicate that prior evaluative judgments and decision commitments persist over time, shaping subsequent evaluations through cognitive continuity rather than independent reassessment. Figure 3 presents a visual summary of these results, complementing the detailed estimates reported in Table 4.

As summarized in Table 7, the hypothesis testing results partially support H1, indicating that the importance of certain hotel choice attributes—particularly room quality and visitor reviews—increased over time. Additionally, the satisfaction–platform loyalty intentions relationship (H2 and H3) was strongly supported, demonstrating that satisfaction exerts an increasingly influential role in shaping continued OTA usage intentions across repeated consumption episodes. Finally, the carryover effects of satisfaction and platform loyalty intentions (H4 and H5) were confirmed, indicating that consumers’ prior evaluations persistently influence subsequent judgments and platform-related behavioral intentions. Taken together, these findings reveal a coherent pattern of experience-driven evaluative reweighting, judgment reinforcement, and cognitive continuity in repeated decision-making.

**Figure 4:**
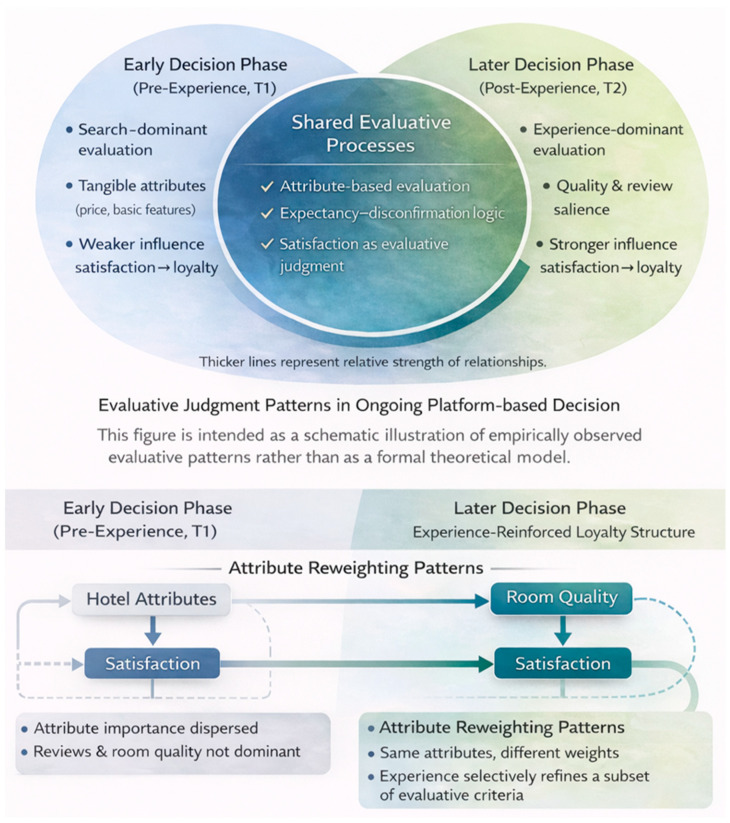
Illustrative Comparison of Evaluative Judgment Patterns Before and After Experience. Note. This figure is intended as a schematic illustration of empirically observed evaluative patterns rather than as a formal theoretical model. Satisfaction is measured at both time points (T1 and T2) to capture changes over time. The direct lines = direct effects; The dashed lines = indirect effects.

Figure 4 provides a schematic illustration of empirically observed evaluative judgment patterns across repeated OTA-mediated hotel choice decisions. As illustrated in Figure 4 both the early (pre-experience, T1) and later (post-experience, T2) decision phases share a set of common evaluative processes, in which satisfaction functions as an integrative evaluative judgment linking attribute-based assessments to continued platform usage intentions. Across both phases, consumers rely on attribute-based evaluation within an OTA-mediated decision environment, and satisfaction is formed through an expectancy–disconfirmation logic.

Figure 4 highlights how the relative strength and organization of these evaluative processes change as experience accumulates. In the early decision phase (T1), evaluations are predominantly search-oriented, with attribute importance relatively dispersed and the satisfaction–platform loyalty relationship comparatively weak. In the later decision phase (T2), accumulated consumption experience leads to selective attribute reweighting, with room quality and online reviews emerging as more salient inputs to satisfaction. As a result, satisfaction exerts a substantially stronger influence on subsequent platform loyalty intentions.

Notably, this comparison indicates that changes in platform loyalty intentions arise not from the emergence of new evaluative mechanisms, but from experience-driven reinforcement and reorganization of existing evaluative criteria. By contrasting stability in core evaluative processes with changes in their relative strength and configuration, Figure 4 underscores the dynamic nature of evaluative cognition in experience-based decision contexts and illustrates why a longitudinal perspective is essential for understanding how satisfaction-based judgments stabilize over time.

## 6. Discussion

This study examined how consumers form and strengthen platform loyalty intentions over time in OTA-mediated hotel choice, focusing on the dynamic evolution of satisfaction and attribute importance across consumption stages. Drawing on Mittal et al.’s (1999) consumption system approach and Oliver’s (2010) satisfaction cycle, we show that platform loyalty does not emerge as a static outcome of isolated evaluations. Instead, individuals develop it through a temporally embedded process of evaluative judgment as they accumulate decision experience.

By adopting a longitudinal design, this study moves beyond the dominant cross-sectional perspective in electronic commerce and hospitality research and advances a behavioral science view of loyalty as an adaptive cognitive process rather than a fixed attitudinal state. This perspective aligns with the decisions-from-experience literature, which suggests that experiential learning and accumulated feedback from prior outcomes play a greater role in shaping repeated decisions than stable preference structures (Erev & Haruvy, 2013; Hertwig & Erev, 2009).

The results show that satisfaction exerts a stronger effect on platform loyalty intentions after consumers experience the hotel, indicating that satisfaction becomes a more powerful driver of continued OTA use as individuals transition from search-based to experience-based evaluation. From a behavioral science perspective, satisfaction increasingly functions as a source of decision confidence and cognitive reliance in repeated decision-making. Although prior studies consistently report a positive association between satisfaction and loyalty, they typically assume this relationship remains temporally stable.

In contrast, the present findings reveal that the satisfaction–platform loyalty linkage intensifies over time, indicating that repeated experience reinforces the role of evaluative judgments in guiding future decisions. This pattern supports the core proposition of decisions-from-experience research: individuals increasingly rely on prior outcomes when forming new decisions and use past evaluations as a basis for future choice (Erev & Haruvy, 2013; Hertwig & Erev, 2009). In this sense, satisfaction may function as a learned evaluative signal that guides future decisions through reinforcement and memory-based reliance.

Beyond experiential learning, the findings indicate that positive service outcomes strengthen platform loyalty because of the experience and because consumers perceive that the platform successfully facilitated a good decision. When platform-provided information, such as reviews, ratings, and recommendations, accurately predicts experienced quality, consumers attribute positive outcomes to the platform. This attribution strengthens trust and reliance. This interpretation aligns with expectation confirmation and search efficiency mechanisms, through which platforms gain loyalty by reducing uncertainty and enabling more effective choices.

The study also reveals that platform loyalty develops through attribute reweighting rather than through the introduction of new evaluative criteria. Specifically, room quality and online reviews become increasingly salient after consumption, whereas other attributes remain stable or decline in importance. Experience reorganizes existing evaluative hierarchies by selectively reinforcing certain criteria. This finding supports adaptation-level theory (Mittal et al., 1999; Zhi & Ha, 2024) and extends the consumption system framework by demonstrating how experiential learning dynamically recalibrates attribute-level evaluations in platform-mediated environments.

At first glance, the increased influence of online reviews after direct experience appears inconsistent with Bayesian learning models, which predict that private information reduces reliance on public information. However, from a behavioral perspective, cognitive dissonance processes explain this pattern. After making a decision, consumers often seek confirmation that they chose appropriately and turn to others’ opinions for validation (Festinger, 1962; Harmon-Jones & Mills, 1999). In this context, online reviews provide an external reference point that helps individuals assess whether their experience aligns with broader evaluations. Thus, rather than replacing reviews, direct experience may increase their importance as consumers use them to verify and justify their decisions. This interpretation suggests that consumers rely more heavily on reviews as part of a validation process that promotes consistency between personal judgments and external information.

Finally, the carryover effects of satisfaction and platform loyalty intentions demonstrate that consumer evaluations operate intertemporally. Prior evaluative judgments persist and shape subsequent evaluations, reflecting cognitive continuity instead of independent reassessment across decision episodes. These findings provide empirical support for recursive loyalty formation mechanisms proposed in Oliver’s (2010) satisfaction cycle and Johnson et al.’s (2006) loyalty evolution framework, which researchers have rarely tested longitudinally in platform-based service settings.

Overall, this study contributes to behavioral science by showing how evaluative judgments stabilize and reorganize through experience-based updating. By foregrounding time, learning, and attribute reweighting, the findings underscore the importance of longitudinal approaches for understanding how evaluative cognition stabilizes and guides behavior in experience-based decision contexts, including online travel platforms. Accordingly, readers should interpret our findings as shifts in the relative salience of evaluative criteria rather than precise parameter changes, particularly given our use of single-item attribute measures. This interpretation also aligns with research on attitude stability and memory-based evaluation, which shows that accumulated experience increases individuals’ reliance on prior evaluations when forming subsequent judgments.

### 6.1. Theoretical Implications

This study contributes to the literature on platform-based loyalty by providing a longitudinal, process-oriented account of how platform loyalty intentions evolve in OTA-mediated hotel choice. Rather than reiterating descriptive findings, we advance three theoretical contributions that extend satisfaction-based loyalty theories by conceptualizing loyalty as a temporally evolving evaluative judgment instead of a static attitudinal outcome.

First, this study clarifies the temporal dynamics of the satisfaction–platform loyalty relationship by demonstrating that its strength changes over time. Although prior hospitality and electronic commerce research consistently documents a positive relationship between satisfaction and loyalty, it has largely relied on cross-sectional evidence that obscures how this relationship develops (Pee et al., 2019; So et al., 2025). We show that satisfaction exerts a significantly stronger influence on platform loyalty intentions after consumers accumulate actual consumption experience. This finding highlights time as an important dimension in loyalty formation and suggests that the behavioral meaning of satisfaction evolves as evaluative judgments stabilize through experience (Petrocelli et al., 2010). In platform-mediated contexts, satisfaction increasingly functions as a cognitive signal of decision confidence rather than solely as a transient post-consumption reaction.

Second, this study identifies attribute reweighting as a key mechanism through which loyalty dynamics unfold during the transition from pre-consumption expectations to post-consumption experience. Our findings show that loyalty evolves because consumers adjust the relative importance of existing hotel choice attributes, particularly room quality and online reviews. This result extends adaptation-level theory (Helson, 1964) by illustrating how experiential learning reorganizes evaluative priorities over time and strengthens the link between satisfaction and future decision commitment. Instead of assuming fixed attribute importance, this study demonstrates that consumers adapt evaluative standards through experience and learn which cues deserve greater cognitive weight in experience-based decision contexts.

Third, this study extends satisfaction-based loyalty theories to platform-mediated consumption systems by clarifying their boundary conditions. Mittal et al.’s (1999) consumption system framework conceptualizes repeated consumption around a focal service provider and suggests that satisfaction’s diagnostic value may weaken as novelty fades. In contrast, our findings show that OTA-mediated contexts operate differently. In platform-based systems, the platform serves as a stable evaluative anchor, whereas individual hotels function as interchangeable alternatives within that system. As consumers accumulate experience, they selectively align evaluative priorities with attributes that the platform enables them to assess effectively, such as perceived room quality and online reviews.

We interpret these findings within the scope of our two-wave design, which captures the transition from pre-consumption expectations to post-consumption experience within a focal OTA-mediated decision episode. Over time, satisfaction appears to signal confidence in the platform’s decision-structuring capability rather than diminishing informational value, which strengthens the satisfaction–platform loyalty relationship. By empirically examining carryover effects in satisfaction and platform loyalty intentions, we extend satisfaction-based frameworks to platform-mediated decision contexts and underscore the importance of distinguishing platform-level evaluative commitment from firm-level loyalty in behavioral and electronic commerce research.

### 6.2. Managerial Implications

The findings of this study offer several managerial implications for OTAs and platform managers seeking to support sustained platform usage through hotel choice experiences. Rather than viewing hotel attributes as static drivers of satisfaction, the results suggest that platform design and information presentation may benefit from aligning with how evaluative priorities evolve as users accumulate experience. From this perspective, managerial action is less about maximizing immediate persuasion and more about supporting decision environments that facilitate evaluative learning over time.

First, the results suggest that OTAs may benefit from differentiating information presentation strategies according to users’ experience levels. Experiential and hedonic attributes (e.g., leisure activities and food and beverage offerings) appear to play a more prominent role during initial hotel selection, whereas core functional attributes—particularly room quality and online reviews—become increasingly salient after consumption. This pattern suggests that early-stage users may rely on broader exploratory cues, while more experienced users may increasingly attend to attributes that facilitate performance verification and expectation calibration. Thus, OTA interfaces could consider adopting more adaptive presentation strategies, emphasizing experiential variety for less experienced users while foregrounding reliability-related cues for repeat users, thereby supporting more informed satisfaction formation across decision episodes within the observed context.

Second, the increasing salience of online reviews for repeat users underscores the role of OTAs as decision-support and trust-calibration environments rather than mere transaction platforms. As users accumulate experience, peer-generated content becomes a key resource for evaluating hotel performance and for reinforcing confidence in future platform-mediated choices. From a managerial standpoint, this suggests that investing in mechanisms that enhance the diagnostic clarity of reviews may be beneficial—such as verified-stay indicators, reviewer segmentation, or temporally organized summaries—not to persuade users, but to facilitate more accurate performance inference and expectation adjustment.

Third, attributes whose importance remains relatively stable across consumption episodes—such as hotel reputation, access to local information, and concierge-related services—appear to function as baseline evaluative anchors rather than as dynamic differentiation cues. For OTAs, this suggests that maintaining consistent, transparent, and standardized presentation of these attributes may help support evaluative continuity during the transition from pre-consumption expectations to post-consumption experience. Rather than serving as sources of competitive advantage, these stable attributes may operate as baseline requirements, where their absence may disrupt satisfaction and trust, while their consistent presence may support ongoing platform use. Ensuring uniform information quality and dependable access to such baseline cues may therefore contribute to stabilizing users’ evaluative frameworks over time.

### 6.3. Limitations and Future Research Directions

Despite its contributions to understanding platform loyalty formation in OTA-mediated hotel choice, this study has several limitations that suggest avenues for future research. In particular, these limitations point not to weaknesses in the core findings, but to opportunities for further elaborating the temporal and cognitive mechanisms underlying repeated decision-making.

First, the longitudinal design was limited to two observation points, which constrains the ability to capture more complex nonlinear trajectories in satisfaction and platform loyalty development. Although the two-wave design allowed for the examination of temporal change and carryover effects, future studies incorporating three or more time points could more fully model how evaluative judgments initially adapt, subsequently stabilize, and potentially decay over extended decision cycles. Multi-wave designs would enable researchers to distinguish between short-term recalibration effects and longer-term patterns of judgment consolidation or erosion in platform usage.

Second, this study focused on attribute-level evaluations as independent predictors of satisfaction, without explicitly modeling higher-order attribute structures. Future research could extend this approach by grouping attributes into theoretically meaningful categories (e.g., functional quality, experiential value, informational reliability) or by employing hierarchical or multilevel modeling techniques. Such approaches would allow scholars to examine how different layers of evaluative cognition interact over time, offering a more integrative account of how attribute-level judgments aggregate into stable evaluative standards.

Third, although procedural and statistical remedies were employed to minimize common method bias, the exclusive reliance on self-reported survey data remains a limitation. Future studies could strengthen causal inference by integrating survey measures with behavioral indicators, such as actual booking histories, search sequences, or repeat usage logs obtained from OTA platforms. Linking subjective evaluations with observable behavioral traces would enable a more precise mapping between evaluative cognition and realized decision behavior across time.

Fourth, we measured platform loyalty as self-reported intention rather than as observed behavioral outcomes. Although prior research widely used intention as a proxy for future behavior, intention may not fully capture actual behavioral loyalty in real-world settings. Therefore, readers should interpret our findings as reflecting attitudinal dynamics rather than realized behavioral repetition. Future research could extend this work by incorporating behavioral data, such as actual booking records, to examine whether similar patterns hold for observed loyalty behavior.

Fifth, we used single-item indicators to measure specific hotel choice attributes. Researchers commonly adopt this approach when assessing concrete and easily interpretable service features, particularly when attributes are unidimensional and directly observable (Bergkvist & Rossiter, 2007). However, single-item measures do not allow for the assessment of internal consistency reliability and may introduce additional measurement error. Consequently, some degree of noise may affect the estimated attribute effects. Readers should therefore interpret changes in attribute weights as shifts in relative evaluative salience rather than as precise quantitative differences in parameter estimates. Future research could strengthen these findings by aggregating related attributes into theoretically meaningful categories and examining whether similar patterns of evaluative reweighting emerge.

Sixth, the empirical context of this study was limited to the South Korean hotel market, which may constrain the generalizability of the findings. Cultural characteristics such as relational norms, uncertainty avoidance, and technology adoption tendencies may influence how satisfaction translates into platform loyalty. Future research should therefore examine the proposed model across different cultural and institutional settings, as well as across other platform-based service domains (e.g., ride-sharing, food delivery, or accommodation-sharing platforms), to assess the boundary conditions under which evaluative reweighting and judgment reinforcement processes operate.

Finally, our further limitation is the absence of objective price information in the empirical model. From an economic perspective, actual price is a fundamental component of the consumer utility function and plays a critical role in shaping decision-making. Although perceived room price was included as an evaluative attribute, it may not fully capture the influence of actual price on consumer evaluations.

In this study, we attempted to mitigate potential omitted variable bias by restricting the sample to 4- and 5-star hotels and by incorporating contextual control variables, such as hotel size and location. While these strategies help reduce unobserved heterogeneity, they represent a methodological compromise and do not fully substitute for objective price measures. As a result, the estimated effects of experiential attributes, particularly core attributes such as hotel room quality and online reviews, may be partially confounded with unobserved price variation across hotels.

Given the survey-based nature of the dataset, it was not feasible to incorporate transactional price data in the present analysis. Future research is encouraged to utilize objective pricing information from platform-based or transactional data sources to validate and extend the present findings. Such approaches would enable more precise estimation of attribute effects and help address concerns related to omitted variable bias.

## Figures and Tables

**Table 1:** Measures and Factor Loading by Time Period.

Construct	Outer Longing		AVE	
	T1	T2	T1	T2
*Satisfaction with OTA-mediated hotel choice experience* (CR: T1 = 0.87; T2 = 0.85).			0.68	0.65
The experience of staying at the hotel booked through this OTA met or exceeded what I expected.	0.78	0.87		
My decision to choose this hotel through the OTA turned out to be a wise one based on its performance.	0.84	0.74		
Overall, I am satisfied (would be satisfied) with the hotel experience relative to my expectations formed via the OTA.	0.86	0.81		
*Platform loyalty intentions* (CR: T1 = 0.90; T2 = 0.91).			0.75	0.76
If I need to book a hotel in the future, I intend to use this OTA again.	0.87	0.88		
This OTA would be my first choice for future hotel bookings.	0.89	0.87		
I would recommend this OTA to others for hotel reservations.	0.83	0.85		

Note. CR = Composite reliability.

**Table 2:** Means and Reliabilities for Scales by Time Period.

Variable	T1: Mean (SD)	T2: Mean (SD)	Alpha T1 (T2)
Satisfaction	4.14 (0.65)	4.24 (0.57)	0.76 (0.73)
Platform loyalty intentions	4.05 (0.83)	4.25 (0.72)	0.84 (0.83)
Hotel room quality	4.10 (0.53)	4.30 (0.56)	
Safety and security	4.18 (0.59)	4.53 (0.54)	
Food and beverage	4.16 (0.61)	4.32 (0.57)	
Leisure activities	3.93 (0.77)	4.14 (0.71)	
Perceived room price	4.11 (0.76)	4.33 (0.66)	
Hotel reputation	3.95 (0.77)	4.10 (0.75)	
Level of staff politeness	3.90 (0.72)	4.08 (0.73)	
Cleanliness	3.91 (0.80)	4.14 (0.69)	
Relaxing lounge or bar availability	3.92 (0.75)	4.15 (0.68)	
Parking	3.68 (0.77)	3.88 (0.80)	
Various information is provided around the hotel	4.10 (0.65)	4.22 (0.63)	
Luggage service	4.03 (0.60)	4.16 (0.54)	
Concierge service	3.79 (0.77)	3.98 (0.80)	
Swimming pool	4.09 (0.66)	4.25 (0.67)	
Visitor reviews	4.04 (0.69)	4.24 (0.69)	

**Table 3:** Attrition Analysis at T1.

Variable	Retained (n = 481) Mean (SD)	Dropped (n = 240) Mean (SD)	t-Value	*p*-Value
Satisfaction (T1)	4.16 (0.62)	4.10 (0.65)	0.95	0.34
Platform loyalty intentions (T1)	4.08 (0.80)	4.02 (0.82)	0.88	0.38
Hotel room quality	4.12 (0.53)	4.08 (0.55)	0.74	0.46
Visitor reviews	4.05 (0.69)	4.00 (0.71)	0.81	0.42
Perceived room price	4.14 (0.76)	4.09 (0.78)	0.69	0.49
Age	33.2 (7.4)	32.8 (7.6)	0.61	0.54

Note: All variables are measured at T1. None of the differences between the retained and dropout samples are statistically significant.

**Table 4:** Discriminant Validities.

**Fornell & Larcker Criterion**				
	1	2	3	4
1. Satisfaction (T1)	*0.82*			
2. Platform loyalty intentions (T1)	0.23	*0.86*		
3. Satisfaction (T2)	0.01	0.02	*0.81*	
4. Platform loyalty intentions (T2)	0.02	0.11	0.62	*0.87*
**Heterotrait–Monotrait Ratio (HTMT)**				
1. Satisfaction (T1)				
2. Platform loyalty intentions (T1)	0.27			
3. Satisfaction (T2)	0.05	0.06		
4. Platform loyalty intentions (T2)	0.04	0.13	0.68	

Note: Italic numbers are the square root of AVE.

**Table 5:** Attribute Weight Shifts over Time.

Attribute	Weight at T1	Weight at T2	Difference (Δ)	Significant?
Hotel room quality	0.01	0.18	0.17	Yes
Safety and security	0.02	0.06	0.04	Yes
Food and beverage	0.13	0.08	−0.05	Yes
Leisure activities	0.09	0.03	−0.06	Yes
Perceived room price	0.01	0.14	0.13	Yes
Hotel reputation	0.05	0.05	-	No
Level of staff politeness	0.05	0.07	0.02	No
Cleanliness	0.06	0.02	−0.04	Yes
Relaxing lounge or bar availability	0.05	0.09	0.04	Yes
Parking	0.02	0.01	−0.01	No
Various information is provided around the hotel	0.04	0.04	-	No
Luggage service	0.03	0.16	0.13	Yes
Concierge service	0.11	0.11	-	No
Swimming pool	0.01	0.03	0.02	No
Hotel visitor reviews	0.07	0.29	0.22	Yes

**Table 6:** Within-Subject Difference-Score Analysis for Changes in Satisfaction and Platform Loyalty Intentions.

Predictor	Model 1: ΔLOY (β, t)	Model 2: ΔSAT (β, t)
Δ Satisfaction (ΔSAT)	0.26 *** (3.08)	-
Δ Hotel room quality	-	0.18 ** (2.76)
Δ Visitor reviews	-	0.27 *** (3.62)
Δ Perceived room price	-	0.11 † (1.74)
Δ Luggage service	-	0.09 (1.51)
R^2^	0.07	0.16
N	481	481

Note. We computed Δ variables as the difference between T2 and T1 (T2 − T1) using latent variable scores extracted from PLS-SEM. We used bootstrapping with 5000 resamples to estimate significance. † *p* < 0.10, ** *p* < 0.01, *** *p* < 0.001.

**Table 7:** Summary of Hypothesis Testing Results.

Hypothesis	Statement	Result	Supporting Evidence
H1	The relative weights of evaluative criteria evolve over time as individuals accumulate experience through OTA-mediated decisions.	Partially supported	Six attributes increased, three decreased, six unchanged.
H2	Satisfaction at T1 has a direct and positive effect on platform loyalty intentions at T1, reflecting its role as an initial evaluative judgment in ongoing decision-making.	Supported	Significant path coefficient (β = 0.23, *p* < 0.05).
H3	The effect of satisfaction on platform loyalty intentions strengthens over time (T2) as evaluative criteria stabilize and decision confidence increases through experience-driven reweighting.	Supported	Path coefficient increased from β = 0.23 (T_1_) to β = 0.61 (T_2_); Δβ significant (*p* < 0.05).
H4	Prior satisfaction at T1 positively affects satisfaction at T2, reflecting the persistence of evaluative judgments through cognitive carryover.	Supported	Significant carryover effect (β = 0.06, *p* < 0.05).
H5	Prior platform loyalty intentions at T1 positively affect platform loyalty intentions at T2, indicating continuity in decision commitment between the pre-consumption and post-consumption phases.	Supported	Significant carryover effect (β = 0.10, *p* < 0.05).

Note. Results derived from longitudinal PLS-SEM analysis (Figure 1, Figure 2 and Figure 3). H1 received partial support because only 9 of 15 attribute weights showed significant temporal change, while H2–H5 were fully supported.

## Data Availability

The data presented in this study are available on request from the corresponding author due to restrictions on external exposure of data.

## Appendix A. Measurement Items

All measurement items used in this study are reported below. All items were measured using a 5-point Likert scale (1 = strongly disagree, 5 = strongly agree).

### Appendix A.1. Hotel Choice Attributes

Hotel choice attributes were measured using single-item indicators based on prior research.

**Attribute**	**Measurement Statement**	**Source(s)**
Hotel room quality	The size of the room is generous.	Albayrak and Caber (2015)
Safety and security	I felt safe during my hotel stay.	Law and Hsu (2005)
Food and beverage	The hotel’s food and beverage service was satisfactory.	Chou and Chen (2014); J. Kim et al. (2019)
Leisure activities	The hotel provided enjoyable leisure activities.	Bodet et al. (2017)
Perceived room price	The room price was reasonable for the quality of service provided.	Srivastava and Kumar (2021)
Reputation	The hotel has a good reputation among guests.	Francesco and Roberta (2019)
Staff politeness	The hotel staff were polite and professional.	Caber and Albayrak (2014)
Cleanliness	I like the cleanliness of the room.	Albayrak and Caber (2015)
Lounge/bar availability	The hotel offered a relaxing lounge or bar area.	Bodet et al. (2017)
Parking	Parking facilities were convenient and easily accessible.	J. Kim et al. (2020)
Information provision	The hotel provided useful information about nearby attractions.	Qu et al. (2000)
Luggage service	The luggage service was prompt and reliable.	Loureiro and Kastenholz (2011)
Concierge service	The concierge service was helpful and efficient.	Caber and Albayrak (2014)
Swimming pool	The swimming pool was clean and well-maintained.	Srivastava and Kumar (2021)
Visitor reviews	Visitor reviews accurately reflected the hotel’s quality.	J. M. Kim et al. (2023)

### Appendix A.2. Customer Satisfaction

Hotel choice attributes were measured using single-item indicators based on prior research.

Customer satisfaction was measured using three items adapted from prior research.

- I am satisfied with my hotel choice.
- My decision to choose this hotel was a wise one.
- The hotel met my expectations.

### Appendix A.3. Platform Loyalty Intentions

Platform loyalty intentions were measured using three items adapted from Johnson et al. (2006) and El-Adly (2019).

- I intend to continue using this OTA for future hotel bookings.
- I will choose this OTA over other platforms when booking hotels.
- I would recommend this OTA to others.

In this study, loyalty is operationalized as platform loyalty intentions rather than observed behavioral outcomes.
